# Supplementary material for: Long-term cost-effectiveness of interventions for obesity: A mendelian randomisation study
Source: PLoS Med. 2021 Aug 27;18(8):e1003725. doi: 10.1371/journal.pmed.1003725 (PMC8437285; doi:10.1371/journal.pmed.1003725)
Supplement: S1 Text — (DOCX) [file pmed.1003725.s001.docx]

# 1. Description of Studies Estimating the Cost-effectiveness of Interventions

### Randomised Controlled Trials

In randomised controlled trials (RCTs) of BMI interventions, participants are randomised to either receive the intervention or some comparator, and followed-up to determine both the healthcare costs and QALYs over time, **Fig 1, panel a**. This ensures exchangeability of the intervention and control groups (in well-conducted trials), and complete data can in principle be measured. The main strength of RCTs is that, when well conducted, the results give causal estimates of the effect of the intervention on the studied outcomes. However, RCTs are expensive, follow-up times are often limited in duration, randomisation of large numbers of individuals may be difficult, expensive, and may not be generalisable to the population of interest, and there may be bias if adherence is low or there is missing follow-up.

A 2009 systematic review combined data from 23 RCTs for bariatric surgery, although an economic evaluation was not necessarily performed in each RCT [1]. Of these RCTs, the median number of participants was 60 (interquartile range [IQR] 50 to 90, range 30 to 155), and the median follow-up time was 24 months (IQR 12 to 36 months, range 12 to 84 months). Only 3 RCTs had a follow-up time of at least 5 years, with 257 participants in total [2–4]. One of these RCTs completed 10 years of follow-up, with 43 remaining participants [5].

### Cohort Studies

In cohort studies of BMI interventions, a group of people who underwent an intervention to reduce body mass index (BMI) are compared against a group of people who did not receive the intervention, in terms of both QALYs and healthcare costs, **Fig 1, panel b**. The non-intervention group is typically selected to be as similar to the intervention group as possible, with the intended sole difference being in whether they received the intervention. The follow-up time is determined by the data.

The main strength of cohort studies is that many healthcare costs can potentially be captured, for example using large routinely collected datasets. The main limitation is that the group that did not receive the intervention is unlikely to be exchangeable with the group that did, meaning there is a risk of bias from confounding and reverse causation. The follow-up time could also be limited depending on the data available and the novelty of the intervention.

### Decision Analytic and Simulation Models

Decision analytic models are used to inform systematic decision-making under uncertainty. This generally involves modelling quantitative relationships between variables to define possible consequences associated with an intervention that would flow from the alternative options under evaluation to compare costs and outcomes take a variety of forms. Here, we assume that this modelling will generally involving some degree of simulation to model the uncertainty associated with the evaluation of different options or interventions, although this assumption does not necessarily alter the arguments we make below.

In decision analytic simulation models, a hypothetical target population is simulated over time. As time progresses, each simulated individual can move between several states, typically including different categories of BMI, several health conditions (usually type 2 diabetes, stroke, cancer and cardiovascular diseases), as well as death, **Fig 1, panel c**.

Some simulated individuals receive an intervention for obesity, which alters their risk of moving between the states accordingly, and generally adds risks for post-intervention complications. The risk for any individual moving between states is set at plausible values, usually taken from previous observational studies or datasets. Each state has an associated quality of life and cost, from which the total QALYs and cost per simulated individual can be estimated. The total QALYs and healthcare costs are then compared between the simulated individuals who did and did not receive the treatment to estimate the cost-effectiveness of the intervention. Simulated participants can be followed-up until death. The simulation is often repeated many times to estimate confidence intervals around the estimates.

The main strength of these types of decision analytic simulation studies is in the flexibility of the model to adapt to different populations over a timeframe that is not limited. The key limitations are that the simulations can only account for specified health conditions and therefore are affected by structural uncertainty, and so potentially underestimate the effect of obesity on both QALYs and healthcare costs. These models often rely on observational data to estimate the risk of moving between states and the costs and QALYs associated with each state, and both sets of biases may be biased through confounding, measurement error and reverse causation. The simulations also assume that any benefit of the intervention would act entirely through BMI.

## References

1. Picot J, Jones J, Colquitt JL, Gospodarevskaya E, Loveman E, Baxter L, et al. The clinical effectiveness and cost-effectiveness of bariatric (weight loss) surgery for obesity: A systematic review and economic evaluation. Vol. 13, Health Technology Assessment. 2009.

2. MacLean LD, Rhode BM, Forse RA, Nohr C. Surgery for Obesity - An Update of a Randomized Trial. Obes Surg Incl Laparosc Allied Care. 1995;5(2):145–50.

3. Angrisani L, Lorenzo M, Borrelli V. Laparoscopic adjustable gastric banding versus Roux-en-Y gastric bypass: 5-year results of a prospective randomized trial. Surg Obes Relat Dis. 2007;3(2):127–32.

4. Schouten R, Wiryasaputra DC, Van Dielen FMH, Van Gemert WG, Greve JWM. Long-term results of bariatric restrictive procedures: A prospective study. Obes Surg. 2010;20(12):1617–26.

5. Angrisani L, Cutolo PP, Formisano G, Nosso G, Vitolo G. Laparoscopic adjustable gastric banding versus Roux-en-Y gastric bypass: 10-year results of a prospective, randomized trial. Surg Obes Relat Dis. 2013;9(3):405–13.
