# Supplementary material for: Long-term cost-effectiveness of interventions for obesity: A mendelian randomisation study
Source: PLoS Med. 2021 Aug 27;18(8):e1003725. doi: 10.1371/journal.pmed.1003725 (PMC8437285; doi:10.1371/journal.pmed.1003725)
Supplement: S2 Text — Fig A in S2 Text. Flow chart for study inclusion/exclusion. (DOCX) [file pmed.1003725.s002.docx]

# 2. Inclusion Criteria and Genotyping

## 2.1 Inclusion Criteria

There were 502,506 UK Biobank participants with phenotypic data who had not withdrawn from the study. We excluded participants without white British ancestry, without genetic array data, and those with sex-mismatch (derived by comparing genetic sex and reported sex, n=378) or sex-chromosome aneuploidy (n=652), participants who were outliers in heterozygosity and missing rates (n=968) and participants who were related to a very large number (>200) of other participants from the analysis (n=78,385 in total). We defined white British ancestry as participants who self-reported as “White British” and who had very similar ancestral backgrounds according to the principal component analysis, as described by Bycroft [1]. We excluded participants recruited at the Glasgow and Edinburgh centres as healthcare costs were not available for Scotland (n=31,165). Finally, we excluded participants without a BMI measurement (n=1,224). After these exclusions, 377,227 participants remained, see **Fig A in S2 Text**.

We estimated kinship coefficients using the KING toolset [2] and identified 107,162 pairs of related participants [1]. We applied an in-house algorithm to this list and preferentially identified the participants related to the greatest number of other participants until no related pairs remain. This resulted in the identification of 66,314 participants. These participants were removed for all but the within-family analyses, leaving 310,913 participants in the main and other analyses.

## 2.2 Genotyping

The full data release contains the cohort of successfully genotyped samples (n=488,377). 49,979 individuals were genotyped using the UK BiLEVE array and 438,398 using the UK Biobank axiom array. Pre-imputation QC, phasing and imputation are described elsewhere [1]. In brief, prior to phasing, multiallelic SNPs or those with MAF ≤1% were removed. Phasing of genotype data was performed using a modified version of the SHAPEIT2 algorithm [3]. Genotype imputation to a reference set combining the UK10K haplotype and HRC reference panels [4] was performed using IMPUTE2 algorithms [5]. The analyses presented here were restricted to autosomal variants within the HRC site list using a graded filtering with varying imputation quality for different allele frequency ranges. Therefore, rarer genetic variants are required to have a higher imputation INFO score (Info>0.3 for minor allele frequency (MAF) >3%; Info>0.6 for MAF 1-3%; Info>0.8 for MAF 0.5-1%; Info>0.9 for MAF 0.1- 0.5%) with MAF and Info scores having been recalculated on an in house derived ‘European’ subset.

Further information on the MRC-IEU quality control of UK Biobank genetic data is available online [6].


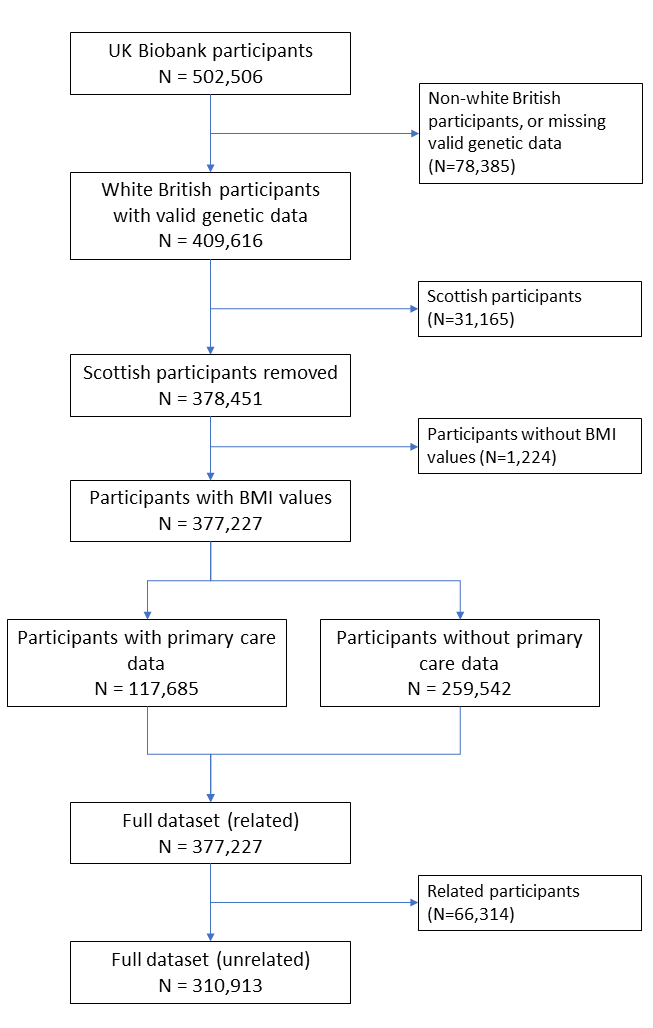


**Fig A:** Flow chart for study inclusion/exclusion

**Legend:** Flow chart for study inclusion/exclusion

## References

1. Bycroft C, Freeman C, Petkova D, Band G, Elliott LT, Sharp K, et al. Genome-wide genetic data on ~500,000 UK Biobank participants. bioRxiv [Internet]. 2017;166298. Available from: https://www.biorxiv.org/content/early/2017/07/20/166298

2. Manichaikul A, Mychaleckyj JC, Rich SS, Daly K, Sale M, Chen WM. Robust relationship inference in genome-wide association studies. Bioinformatics. 2010;26(22):2867–73.

3. O’Connell J, Sharp K, Shrine N, Wain L, Hall I, Tobin M, et al. Haplotype estimation for biobank-scale data sets. Nat Genet. 2016;48(7):817–20.

4. Huang J, Howie B, McCarthy S, Memari Y, Walter K, Min JL, et al. Improved imputation of low-frequency and rare variants using the UK10K haplotype reference panel. Nat Commun. 2015;6.

5. Howie B, Marchini J, Stephens M. Genotype Imputation with Thousands of Genomes. G3&amp;#58; Genes|Genomes|Genetics [Internet]. 2011;1(6):457–70. Available from: http://g3journal.org/lookup/doi/10.1534/g3.111.001198

6. Mitchell, R., Hemani, G., Dudding, T., Corbin, L., Harrison, S., Paternoster L. UK Biobank Genetic Data: MRC-IEU Quality Control, version 2 - Datasets - data.bris [Internet]. data.bris. 2018. Available from: https://data.bris.ac.uk/data/dataset/1ovaau5sxunp2cv8rcy88688v
