# Supplementary material for: Long-term cost-effectiveness of interventions for obesity: A mendelian randomisation study
Source: PLoS Med. 2021 Aug 27;18(8):e1003725. doi: 10.1371/journal.pmed.1003725 (PMC8437285; doi:10.1371/journal.pmed.1003725)
Supplement: S3 Text — Supplementary methods, including 3.1: Estimation of health-related quality of life, 3.2: Dealing with missing data, 3.3: Sensitivity analyses, 3.4: Policy analyses, and 3.5: Worked example of a policy analysis. (DOCX) [file pmed.1003725.s003.docx]

# 3. Supplementary Methods

## 3.1 Estimation of Health-Related Quality of Life

We predicted health-related quality of life for all participants using the results from a study by Sullivan et al., which catalogued health-related quality of life across 240 health conditions [1]. Briefly, in the Sullivan study, community-based UK preferences were applied to EQ-5D-3L descriptive questionnaire responses in the US-based Medical Expenditure Panel Survey. The marginal disutility of each of 240 ICD-9 coded health conditions were estimated with multiple regression, with age, sex, ethnicity, education level, income and number of comorbid health conditions as covariates. We mapped each of the ICD-9 health condition codes to ICD-10 codes, as the majority of HES data in UK Biobank used ICD-10 codes. We also mapped ICD-9 codes to read v2 and v3 codes used in primary care, see **S2 Table** for a list of all ICD and read codes used. Finally, we mapped education level from the US to the UK education system, where “high school” = A levels and below, “Bachelor’s degree” = university or college degree, and “other degree” = National Vocational Qualification or equivalent, or other professional qualification.

We used the Sullivan regression results to predict health-related quality of life for each participant in UK Biobank daily from recruitment to 31 March 2017. To do this, we coded each of the 240 health conditions in the catalogue as dummy variables for every day after recruitment, coded as 1 if the participant had the corresponding ICD-9, ICD-10, read v2 or read v3 code in a hospital episode or primary care event at or before that day, and 0 otherwise. We used age, sex, education level and the number of comorbid health conditions (from among the 240 health conditions) as covariates; we did not include ethnicity as we only included white British participants, and we assumed all participants had the middle income level. The constant in the model represented the health-related quality of life of a white male participant with no qualifications and no health conditions at age 0. We assumed that once a participant had a chronic health condition, they always had the condition.

Health-related quality of life was predicted by multiplying the value of each covariate against the coefficient of disutility for that variable and summing across all covariables and the constant. Any participants who died had a health-related quality of life of 0 on all days from the date of death. We averaged predicted health-related quality of life between recruitment and the end of follow up (regardless of whether the participant died) to estimate each participant’s quality-adjusted life years (QALYs) per year of follow-up.

## 3.2 Dealing with Missing Data

We used multiple imputation by chained equations to predict both primary care healthcare costs and QALYs (N missing = 214,582, 69%), creating 100 imputed datasets [2]. We also imputed Townsend deprivation index (N missing = 342, 0.1%) and whether the participant had ever smoked (N missing =1,063, 0.3%), as these variables were informative but had some missingness.

For the main analysis, we included the following informative covariables as factor variables (**main imputation model**):

- Centre of recruitment
- Highest qualification:
  - A-levels and below
  - University or college degree
  - Other degree
  - None
- Total household income
  - <£18,000
  - £18,000 to £30,999
  - £31,000 to £51,999
  - £52,000 to £100,000
  - >£100,000

We included the following informative covariables as continuous variables:

- Measured BMI
- PRS-free BMI (BMI with the effect of the PRS for BMI removed)
- Measured BMI multiplied by age
- The PRS for BMI
- The PRS for BMI multiplied by age
- Whether the participant died before 31 March 2017
- Age at recruitment
- Date of recruitment
- 40 principal components
- Total days of follow-up
- Secondary care costs
- QALYs predicted using only HES data
- QALYs predicted using only HES data, and using only cancer, cardiovascular disease, cerebrovascular disease and type 2 diabetes as health conditions.

We analysed the imputed datasets separately, then combined the results from each imputation using Rubin’s rules to give a final estimate for all analyses. For summary statistics of imputed QALYs and total healthcare costs, we reported the median of the medians in the imputed datasets, and the median of the interquartile ranges in the imputed datasets. For analyses using imputed data, we reported the median P value for endogeneity with the interquartile range (IQR) from all imputations.

To preserve consistency between the imputation of missing data and the sensitivity analyses, we imputed additional datasets with different covariables. In addition to the covariables in the main imputation model, we included five age categories (<50 years, 50-54 years, 55-59 years, 60-64 years and 65+ years) and whether the participant had a normal (<25 kg/m^2^), overweight (25-29.99 kg/m^2^) or obese (30+ kg/m^2^) PRS-free BMI for the analyses within each category of age and PRS-free BMI (but did *not* include continuous age or BMI**, Sensitivity analyses b and c, categorical imputation model**), the fiftieth of PRS-free BMI as a factor variable for the non-linear MR analyses (but did *not* include PRS-free BMI, **Sensitivity analysis d, BMI quantile imputation model**), and we included all related participants for the within-family analyses (**Sensitivity analysis e, related imputation model**).

## 3.3 Sensitivity Analyses

### a. Mendelian Randomization Sensitivity Analyses

The robustness of Mendelian randomization analyses is reliant on the assumption (amongst others) that the SNPs, and therefore PRS, do not affect the outcome except through the exposure, i.e. the SNPs are not pleiotropic. We tested this assumption by conducting sensitivity analyses using summary data for each SNP in the BMI PRS, including inverse-variance weighted (IVW), MR Egger (an indicator of directional pleiotropy), weighted median, weighted mode and simple mode analyses [3–5]. We also measured Cochran’s Q statistic from the IVW analyses (a measure of heterogeneity in the effects of individual SNPs on the outcome), an indicator of pleiotropy [6] or problems with modelling assumptions [7].

From these analyses, we determined: a) whether the results were consistent with the main Mendelian randomization analysis, which would indicate the results of the main analysis were robust, and b) whether there was evidence of pleiotropy from both the Egger regression constant term and Cochran’s Q statistic. We also visually inspected plots of the sensitivity Mendelian randomization analyses, which would indicate possible bias in the results of the main analysis.

### b. Sex and Age Specific Analyses

Sex and age may both interact with BMI to affect healthcare costs and average QALYs per year. As such, we performed the main Mendelian randomization analysis for men and women separately, and within 5-year age groups. We also performed the main Mendelian Randomization analysis with an interaction term for age and BMI, with the age-BMI interaction term instrumented with an interaction term for age and the PRS for BMI. These results can be used to determine the effect of a change in BMI for any specified age and sex distribution, as shown in the policy analyses.

### c. Testing for non-linear effects of BMI

The effects of an increase in BMI on healthcare costs and average QALYs per year may be larger in overweight and obese participants compared with normal weight participants. Stratifying by observed BMI could bias the analysis, as stratifying on the exposure in an instrumental variable regression can induce collider bias between the genetic instrument and any unmeasured confounders between the exposure and the outcome, since the exposure is on the causal pathway between the instrument and the outcome [8,9]. We can overcome this bias by stratifying on PRS-free BMI, rather than observed BMI, which we estimated by regressing observed BMI on the PRS for BMI, then predicting each participant’s BMI as if they had the mean value of the PRS for BMI. This removes the effect of the PRS on BMI, and so removes the possibility of collider bias.

We then repeated the main mendelian randomization analysis separately in participants with PRS-free BMI levels above 25 kg/m^2^, above 30 kg/m^2^, and between 25 and 30 kg/m^2^, representing overweight and obese participants, obese participants, and overweight participants respectively.

### d. Non-Linear Effects of BMI

In addition to testing for non-linear effects of BMI using categories of PRS-free BMI, we performed non-linear Mendelian randomization to estimate the overall shape of the association between BMI and each outcome. In this, we performed the main Mendelian randomization within fifty quantiles of genetic-free BMI, giving quantile-specific local average causal effects [8,9]. We analysed these estimates using variance weighted least squares (VWLS) models to determine whether there was a change in the effect of BMI on healthcare costs, average QALYs per year, and QALY-costs as BMI increased. We used both linear and cubic models (with respect to the mean PRS-free BMI in each quantile) to describe the shape of the effect of the increase in BMI over the range of PRS-free BMI values.

### e. Within-Family Mendelian Randomization Analysis

Mendelian randomization analyses of unrelated individuals may still be susceptible to bias from family structure, for example, through dynastic effects (when parental genotypes directly affect offspring phenotypes), assortative mating or cryptic relatedness [10,11]. To assess whether any effects of BMI on total healthcare costs and QALYs per year are robust to these family-level effects, we performed non-genetic and Mendelian randomization analyses within siblings. For this analysis, we centred the PRS for BMI (for Mendelian randomization analyses) or observed BMI (for multivariable adjusted analyses) within each set of siblings, and repeated the main analysis with these exposures in only the related participants in UK Biobank. The effect estimates are thus controlled for family-level effects, as only the differences in the BMI PRS between siblings examined. Siblings were identified in UK Biobank as per Bycroft et al. [12] using kinship coefficients from the KING toolset [13].

### f. Estimation of Utilities with Prediction Uncertainty

In the main analysis, we assumed the predicted utilities were estimated without error, i.e. the regression model by Sullivan et al [1] had an R^2^ of 1 and was therefore deterministic of health-related quality of life. However, there was likely prediction error for individuals, which should be considered when analysing the predicted outcomes. The Sullivan paper does not give the mean square error (MSE) of the regression model, which is required to estimate the standard error (SE) of an individual’s predicted health-related quality of life.

The standard error of predicted health-related quality of life for any individual can be estimated using:

$$SE\left( {utility}_{h} \right)=\sqrt{\mathrm{MSE}\left( 1+X_{h}^{T}\left( X^{T}X \right)^{-1}X_{h} \right)}$$

$$X_{h}=\left( 1,X_{h,1},X_{h,2},\ldots,X_{h,k} \right)^{T}$$

where $SE\left( {utility}_{h} \right)$ is the estimate of the standard error of the health-related quality of life for participant $h$, $\mathrm{MSE}$ is the mean square error of the initial regression model (equivalent to the variance of health-related quality of life when R^2^ is 0) and $X$ is a column vector of predictor variables in the regression model.

$X_{h}^{T}\left( X^{T}X \right)^{-1}X_{h}$ is the variance of a mean response, which falls to 0 as N increases. As such, when N is large:

$$X_{h}^{T}\left( X^{T}X \right)^{-1}X_{h}\cong0$$

And therefore, when N is large:

$$SE\left( {utility}_{h} \right)\cong\sqrt{\mathrm{MSE}}$$

Therefore, when the R^2^ of a regression model is 0, the MSE is equivalent to the variance of the outcome. As R^2^ increases, the MSE decreases proportionately, i.e. when R^2^ is 0.25, the MSE decreases by 25%. We estimated the SE of predicted health-related quality of life at 4 different R^2^ values: 0%, 25%, 50% and 75%. The main analysis assumes that the R^2^ value of the initial regression was 100%.

We estimated the SD of health-related quality of life to be 0.20 in the Sullivan study (from the interquartile range, ignoring skew), which as N is large, is approximately equivalent to the root mean square error (RMSE) of predicted health-related quality of life if the R^2^ of the regression model was 0%. The RMSE is therefore reduced to 0.17 at an R^2^ of 25%, 0.14 at 50% and 0.10 at 75% (and 0 at 100%).

To determine whether prediction error could materially affect the main analysis estimates, we assumed that the SE of each health-related quality of life (and thus the average QALYs per year) was equal to the RMSE of the initial regression model at different R^2^ values. This assumes that health-related quality of life can take values higher than 1 (otherwise there would be implications for the SE of utilities close to 1), which in practice can be a reasonable assumption [14]. This also assumes that although health-related quality of life is known to be 0 at death, there is still variance of the estimate. We therefore also estimated the weighted variance for average QALYs per year, setting the variance of health-related quality of life at death to be 0 and averaging variance across all days of follow up.

While we could use variance weighted least squares (VWLS) to account for the uncertainty in predicted average QALYs per year for the multivariable adjusted analyses, Mendelian randomization analyses use instrumental variable regression and we are unaware of any statistical technique that combines VWLS and instrumental variable regression to account for uncertainty in the outcome. As such, we added random normal error (mean 0 and SD equal to the SE of the predicted average QALYs per year for each given R^2^ value) to the predicted average QALYs per year for all participants, creating 100 “imputed” values of QALYs. We repeated the main Mendelian randomization analysis on each “imputed” predicted average QALYs per year and combined the results with Rubin’s rules. As we also imputed QALYs, this meant we estimated 100 combined results (one for each imputed dataset), and we then combined these results again with Rubin’s rules.

The estimates from these analyses incorporate the uncertainty in the prediction of health-related quality of life, so if there is only a minimal increase in the variance of the final Mendelian randomization estimates from including this uncertainty, then we can be more confident that it is not necessary to account for it and the main analysis therefore does not excessively underestimate the variance of each estimate.

### g. Predicting Health-related Quality of Life Using Limited Health Conditions

In addition to using all 240 health conditions as measured by Sullivan et al. [1], we limited the health conditions to those commonly used in simulation models to estimate the cost-effectiveness of interventions for BMI, i.e. cancer, cardiovascular disease, cerebrovascular disease and type 2 diabetes. Multiple ICD-9 codes used by Sullivan et al. related to each of these four health conditions: 19 codes related to cancer, 11 to cardiovascular disease, 4 to cerebrovascular disease, and 1 to type 2 diabetes; which ICD-9 codes we used is detailed in **S2 Table**. We predicted health-related quality of life as in the main analysis, but restricting the covariables to these 35 ICD-9 codes (including when estimating the number of comorbid conditions), as well as the demographic covariables (age, sex, qualifications). We then estimated the average QALYs per year as in the main analysis, with the resulting QALY variable analysed as in the main analysis.

We then compared the estimates for QALYs using all 240 health conditions versus only the limited 35 health conditions to investigate whether the effect of BMI on quality of life extended beyond the conditions usually considered associated with BMI.

## 3.4 Policy Analyses

For all policy analyses, we used data from the Health Survey for England (HSE) in 1993 and 2017 to inform our estimates of the BMI distribution of people in England and Wales [15,16]. The Health Survey for England was designed to provide annual data from nationally representative samples to monitor trends in the nation’s health, and as such the BMI values in this survey should be representative for England and Wales. We applied the analyses to the population of England and Wales in 2017, with an age-distribution from the Office of National Statistics [17].

In each analysis, we used simulation of either individuals or groups to estimate the change in BMI within age and BMI categories due to each intervention. We then multiply the age and BMI category specific BMI changes by the age and BMI category specific Mendelian randomization estimates from **sensitivity analysis** **b** to estimate the effect of the interventions on both QALYs and healthcare costs.

In each simulation, we conducted the following steps:

1. Estimated the effect of the intervention on BMI within age groups (40-49 years, 50-54 years, 55-59 years, 60-64 years and 65-69 years); if the effect of the intervention had uncertainty, the effect in each simulation was drawn from a random normal distribution where the mean is the effect estimate and the SD is the SE of effect estimate (taken from previous studies).
2. Estimated the change in BMI for either the individuals or groups in the simulation given estimated effect of the intervention, split into the change in BMI that occurs over 30 kg/m^2^, between 25 and 30 kg/m^2^, and less than 25 kg/m^2^.
3. Estimated the effect of BMI on QALYs and total healthcare costs, using the Mendelian randomization results from the analyses stratified by age and BMI categories (**S4 Table**); this is drawn from a random normal distribution where the mean is the effect estimate and the SD is the SE of effect estimate.
4. Multiplied the estimated change in BMI in each of the three BMI levels by the BMI- and age-specific effect estimates for QALYs and total healthcare costs for all individuals or groups in the simulation. This is the estimated change in QALYs or costs for each individual or group due to the intervention. If the simulation is for groups, we multiplied the estimated change by the number of participants in the group.
5. Summed the estimated changes across all individuals or groups. This is the estimated change in QALYs or costs across all individuals in the target population.

We repeated the simulation 10,000 times for each analysis; we took the median estimate of these simulations as the final estimate and the 2.5% and 97.5% percentiles as its 95% confidence interval.

To estimate the cost-effectiveness of each intervention with appropriate uncertainty (i.e. accounting for the correlation between QALYs and healthcare costs), we combined the effects of BMI on both QALYs and healthcare costs, assigning a value of £20,000 to the QALYs based on commonly used cost-effectiveness thresholds [18,19]. For this, we subtracted average QALYs per year multiplied by £20,000 from total care costs, creating a new variable called “QALY-costs” in the supplementary tables. We included QALY-costs as an outcome in all main and sensitivity analyses, allowing us to estimate the effect of BMI on combined QALYs and healthcare costs directly, with appropriate uncertainty. We also use cost-effectiveness thresholds of £10,000 and £30,000 in additional analyses. For the examples **a** and **b**, we also include the net monetary benefit, which is QALY-costs minus the cost of the intervention. We include a worked example of this method in **3.5 Worked Example of Policy Analysis** below.

For all examples, we assume that Mendelian randomization is consistent with the stable unit treatment value assumption of causal inference, i.e. that the Mendelian randomization estimates of genetically different BMI are consistent with the effects of an intervention that alters BMI through other means.

### a. Cost-Effectiveness of laparoscopic gastric bypass

In this example, we estimated whether laparoscopic gastric bypass for people aged 40-69 years in England and Wales with a BMI above 35 kg/m^2^ in 2017 is cost-effective as compared to no intervention over 20 years at a cost-effectiveness threshold of £20,000 per QALY and a discount rate for both QALYs and costs of 3.5% per year. We also consider different costs per QALY (£30,000 and £10,000) and different discount rates (1.5% and 0%).

The cost of laparoscopic gastric bypass in the NHS in the UK, including costs of the preoperative assessment, complications, and 5 years of follow-up, was estimated to be £6,347 in 2005 [20], which is £9,549 in 2019 prices after applying inflation from the Office of National Statistics composite price index (average 3.0% per year between 2005 and 2019) [21]. Laparoscopic gastric bypass has been estimated to reduce weight in people with obesity by 25% (95% CI: 22% to 28%), which is the equivalent of reducing BMI by 25%, and this reduction was maintained at both 10 and 20 years of follow-up [22,23]. Our simulation was thus a hypothetical situation where people in England and Wales with a BMI above 35 kg/m^2^ in 2017 received laparoscopic gastric bypass surgery in 2007, the intervention reduced BMI by 25% (with a SD of 1.5%) and lasted for 20 years, with hypothetical follow-up to 2027. We assumed that no participant died from the surgery, nor suffered any long-term complications of surgery that would adversely affect their quality of life, though complications up to 5 years from surgery date were accounted for in the cost of the surgery. Although not all people included in the simulation would be eligible for laparoscopic bariatric surgery (indeed, some would have already had surgery), our aim is to estimate the average effect of surgery on an individual drawn from this population, rather than the effect of treating all eligible people.

We used HSE 2017 data to estimate the mean change in BMI within age groups for all HSE participants aged 40 to 69 years, given the reduction in weight from bariatric surgery. We multiplied the age-specific estimates of the effect on QALYs and costs by the total number of people in those age categories in England and Wales in 2017 [17] to estimate the effect of the intervention nationally. We present the per-person results in the main text, corresponding to per person with a BMI above 35 kg/m^2^ aged 40-69 years in England and Wales in 2017, with results across all people available in a supplementary table.

### b. Cost-Effectiveness of restricting volume promotions for high fat, sugar, and salt products

In this example, we estimated whether restricting volume promotions for high fat, sugar, and salt (HFSS) products in England and Wales was cost-effective over a single year at a cost-effectiveness threshold of £20,000 per QALY. We also consider different costs per QALY (£30,000 and £10,000).

The Government has estimated that ending all volume offers for HFSS products in all retailers and the out-of-home sector will reduce the average Calories consumed by 14 Calories per day for men aged 19-64 years, 13 Calories for men aged 65+ years, 11 Calories for women aged 19-64 years, and 10 Calories for women aged 65+ years [24]. The Department for Health and Social Care Calorie Model estimates the overall weight is reduced on average by 0.042 kg per 1 fewer Calorie consumed per day [25]. We assumed that this intervention had no cost when estimating the net monetary benefit.

We used HSE 2017 data to estimate the mean change in BMI within age groups for all HSE participants aged 40 to 69 years, given the reduction in weight from the intervention on HFSS products. We multiplied the age-specific estimates of the effect on QALYs and costs by the total number of people in those age categories in England and Wales in 2017 [17] to estimate the effect of the intervention nationally. We present the total results across all people aged 40-69 years in England and Wales in 2017 in the main text, with the per-person results available in a supplementary table.

### c. Estimation of the Effect of the Population Change in BMI Between 1993 and 2017

In this example, we estimated the change in QALYs and total healthcare costs between 1993 and 2017 from the change in BMI across the population of England and Wales. The population increased in size between 1993 and 2017, so to ensure comparability of estimates, we estimated the change in each outcome as if the total population and their relative age distribution were the same in 1993 and 2017, and only the BMI profile of the population changed. The estimates are thus a fair reflection of the increased cost due solely to a change in BMI, not including the effects of more people or a changing age distribution.

We used the results of the HSE to estimate the mean BMI within deciles of BMI in both 1993 and 2017, for all participants aged 40 to 69 years [15,16]. The difference in BMI between the mean in each BMI decile represents the change in BMI for each tenth of the population, which allows us to determine whether the increase in BMI between 1993 and 2017 occurred equally across the BMI range, or was concentrated at particular tails of the distribution. We present the per-person results in the main text, corresponding to per person aged 40-69 years in England and Wales in 2017, with results across all people available in a supplementary table.

### d. The Cost of Being Overweight and Obese in 2017

In this example, we estimated the effect of being overweight and obese on QALYs and total healthcare costs in England and Wales in 2017. For this, we used HSE 2017 data to estimate the mean change in BMI required to bring the BMI of all participants down to no more than 25 kg/m^2^, within age groups for all HSE participants aged 40 to 69 years. We multiplied the age-specific estimates of the effect on QALYs and costs by the total number of people in those age categories in England and Wales in 2017 to estimate the effect of the intervention nationally. We present the total results across all people with a BMI above 25 kg/m^2^ aged 40-69 years in England and Wales in 2017 in the main text, with the per-person results available in a supplementary table.

## 3.5 Worked Example of Policy Analysis

In this section, we work through the analysis for policy analysis **d**, the cost of being overweight and obese in 2017.

The HSE 2017 data includes 3,411 participants between the ages of 40 and 69 years. Of these, 2,449 (72%) have a BMI above 25 kg/m^2^. We calculate the amount of BMI each of these participants would need to lose in order to have a BMI of 25 kg/m^2^, split into the amount of BMI lost in the overweight and obese categories. For example, a person with a BMI of 34 kg/m^2^ would lose 4 kg/m^2^ in the obese category, and 5 kg/m^2^ in the overweight category, while a person with a BMI of 28 kg/m^2^ would only lose 3 kg/m^2^ in the overweight category.

We then estimate the number of people in the population each person in HSE represents, assuming HSE is completely representative of the population of England and Wales (within age categories). In 2017, ONS estimated that there were 7,677,215 people aged 40-49 years, 4,129,245 people aged 50-54 years, 3,686,614 people aged 55-59 years, 3,169,875 people aged 60-64 years, and 3,079,548 people aged 65-69 years in England and Wales. In HSE, there were 1,154 people aged 40-49 years, 569 people aged 50-54 years, 620 people aged 55-59 years, 532 people aged 60-64 years, and 536 people aged 65-69 years. Within each age group, we divided the total population in England and Wales by the sample in HSE to estimate the number of people each participant of HSE represents. For example, for 40-49 years, each participant of HSE represented 6,653 people (7,677,215/1,154).

We then multiply the number of people each participant in HSE represented by the amount of BMI lost in the overweight and obese categories, and sum across age groups, to estimate the total amount of BMI that would need to be lost across each age group in England and Wales in 2017 so that no one would have a BMI above 25 kg/m^2^. For example, for people aged 40-49 years, 18,625,840 kg/m^2^ would need to be lost in the overweight category, and 12,012,948 kg/m^2^ would need to be lost in the obese category.

We then estimate the effect on QALYs and total healthcare costs of having this amount of BMI above 25 kg/m^2^ by multiplying the BMI amounts by the age and BMI category specific estimates from the Mendelian randomization analyses. Because the Mendelian randomization effect estimates have uncertainty, we repeat this process 10,000 times, taking the effect estimates from a random normal distribution with a mean of the effect estimate, and a SD of the SE of the estimate. For this example, we will just use the relevant effect estimates as an illustration.

The effect estimate for a unit increase in BMI in the 40-49 year overweight group was -0.35% of a QALY for QALYs and £19.22 for total healthcare costs, and in the obese group was -0.23% of a QALY for QALYs and £12.60 for total healthcare costs. Multiplying these estimates with the total BMI lost in the overweight and obese groups, then adding everything together, gives us estimates of the effect of overweight and obesity in 40-49 years olds in 2017: a reduction of 92,824 QALYs and an increase of £509 million in total healthcare costs. Repeating this process for all age groups, and adding everything together, gives us the following estimates: a reduction of 579,579 QALYs and an increase of £3.58 billion in total healthcare costs.

We repeat these calculations 10,000 times, randomly selecting different age and BMI category specific effect estimates in each simulation, and report the median effect estimates with a 95% confidence interval in each policy example.

## References

1. Sullivan PW, Slejko JF, Sculpher MJ, Ghushchyan V. Catalogue of EQ-5D scores for the United Kingdom. Med Decis Mak. 2011;31(6):800–4.

2. Azur MJ, Stuart EA, Frangakis C, Leaf PJ. Multiple imputation by chained equations: What is it and how does it work? Int J Methods Psychiatr Res. 2011;20(1):40–9.

3. Haycock PC, Burgess S, Wade KH, Bowden J, Relton C, Smith GD. Best (but oft-forgotten) practices: The design, analysis, and interpretation of Mendelian randomization studies. Vol. 103, American Journal of Clinical Nutrition. 2016. p. 965–78.

4. Burgess S, Scott RA, Timpson NJ, Smith GD, Thompson SG. Using published data in Mendelian randomization: A blueprint for efficient identification of causal risk factors. Eur J Epidemiol. 2015;30(7):543–52.

5. Pierce BL, Burgess S. Efficient design for mendelian randomization studies: Subsample and 2-sample instrumental variable estimators. Am J Epidemiol. 2013;178(7):1177–84.

6. Greco M F Del, Minelli C, Sheehan NA, Thompson JR. Detecting pleiotropy in Mendelian randomisation studies with summary data and a continuous outcome. Stat Med. 2015;34(21):2926–40.

7. Hemani G, Bowden J, Davey Smith G. Evaluating the potential role of pleiotropy in Mendelian randomization studies. Hum Mol Genet. 2018;27(R2):R195–208.

8. Staley JR, Burgess S. Semiparametric methods for estimation of a nonlinear exposure-outcome relationship using instrumental variables with application to Mendelian randomization. Genet Epidemiol. 2017;41(4):341–52.

9. Burgess S, Davies NM, Thompson SG. Instrumental variable analysis with a nonlinear exposure-outcome relationship. Epidemiology. 2014;25(6):877–85.

10. Brumpton B, Sanderson E, Hartwig FP, Harrison S, Vie GÅ, Cho Y, et al. Within-family studies for Mendelian randomization: avoiding dynastic, assortative mating, and population stratification biases. bioRxiv [Internet]. 2019;602516. Available from: https://www.biorxiv.org/content/10.1101/602516v1?rss=1&utm_source=dlvr.it&utm_medium=twitter

11. Kong A, Thorleifsson G, Frigge ML, Vilhjalmsson BJ, Young AI, Thorgeirsson TE, et al. The nature of nurture: Effects of parental genotypes. Science (80- ). 2018;359(6374):424–8.

12. Bycroft C, Freeman C, Petkova D, Band G, Elliott LT, Sharp K, et al. Genome-wide genetic data on ~500,000 UK Biobank participants. bioRxiv [Internet]. 2017;166298. Available from: https://www.biorxiv.org/content/early/2017/07/20/166298

13. Manichaikul A, Mychaleckyj JC, Rich SS, Daly K, Sale M, Chen WM. Robust relationship inference in genome-wide association studies. Bioinformatics. 2010;26(22):2867–73.

14. Sullivan PW. Are utilities bounded at 1.0? Implications for statistical analysis and scale development. Vol. 31, Medical Decision Making. 2011. p. 787–9.

15. Office of Population Censuses and Surveys SSD. Health Survey for England, 1993 [Internet]. 2nd Editio. UK Data Service; 1997. Available from: http://doi.org/10.5255/UKDA-SN-3316-1

16. University College London D of E and PH, (NatCen) NC for SR. Health Survey for England, 2017 [Internet]. 2nd Editio. UK Data Service; 2019. Available from: http://doi.org/10.5255/UKDA-SN-8488-2

17. Office For National Statistics. Estimates of the population for the UK, England and Wales, Scotland and Northern Ireland [Internet]. Mid year population estimates. 2019. Available from: https://www.ons.gov.uk/peoplepopulationandcommunity/populationandmigration/populationestimates/datasets/populationestimatesforukenglandandwalesscotlandandnorthernireland

18. National Institute for Health and Clinical Excellence. Process and methods guides; The guidelines manual. Guidel manual London. 2012;(November):147.

19. National Institute for Health and Care Excellence. Guide to the methods of technology appraisal 2013. Natl Inst Heal Care Excell [Internet]. 2013;(April):1–93. Available from: http://www.nice.org.uk/media/D45/1E/GuideToMethodsTechnologyAppraisal2013.pdf

20. Ackroyd R, Mouiel J, Chevallier JM, Daoud F. Cost-effectiveness and budget impact of obesity surgery in patients with type-2 diabetes in three European countries. Obes Surg. 2006;16(11):1488–503.

21. Bank of England. Inflation calculator [Internet]. 2020 [cited 2020 Mar 10]. Available from: https://www.bankofengland.co.uk/monetary-policy/inflation/inflation-calculator

22. Sjöström L, Narbro K, Sjöström CD, Karason K, Larsson B, Wedel H, et al. Effects of bariatric surgery on mortality in Swedish obese subjects. N Engl J Med. 2007;357(8):741–52.

23. Sjöström L. Review of the key results from the Swedish Obese Subjects (SOS) trial - a prospective controlled intervention study of bariatric surgery. Vol. 273, Journal of Internal Medicine. 2013. p. 219–34.

24. Department of Health and Social Care (DHSC). Restricting volume promotions for high fat, sugar, and salt (HFSS) products [Internet]. 2018. Available from: https://assets.publishing.service.gov.uk/government/uploads/system/uploads/attachment_data/file/770705/impact-assessment-for-restricting-volume-promotions-for-HFSS-products.pdf

25. Global and Public Health Group/ Obesity Branch/Childhood Obesity Team/10800. Department of Health and Social Care (DHSC) Calorie Model [Internet]. 2018. Available from: https://assets.publishing.service.gov.uk/government/uploads/system/uploads/attachment_data/file/736417/dhsc-calorie-model-technical-document.pdf
