# Supplementary material for: Long-term cost-effectiveness of interventions for obesity: A mendelian randomisation study
Source: PLoS Med. 2021 Aug 27;18(8):e1003725. doi: 10.1371/journal.pmed.1003725 (PMC8437285; doi:10.1371/journal.pmed.1003725)
Supplement: S4 Text — Fig A in S4 Text. The estimated effect of a 1-kg/m2 increase in BMI on average QALYs per year for each quantile of PRS-free BMI. The solid green line indicates the trend line using cubic variance-weighted least squares. The dashed navy lines indicate the PRS-free BMI category specific estimates from the main mendelian randomisation analysis. The effect estimate for each quantile and its 95% CI is represented by the blue points and red vertical lines. BMI, body mass index; CI, confidence interval; PRS, polygenic risk score; QALY, quality-adjusted life year. Fig B in S4 Text. The estimated effect of a 1-kg/m2 increase in BMI on average total healthcare cost per year for each quantile of PRS-free BMI. The solid green line indicates the trend line using cubic variance-weighted least squares. The dashed navy lines indicate the PRS-free BMI category specific estimates from the main mendelian randomisation analysis. The effect estimate for each quantile and its 95% CI is represented by the blue points and red vertical lines. BMI, body mass index; CI, confidence interval; PRS, polygenic risk score; QALY, quality-adjusted life year. (DOCX) [file pmed.1003725.s004.docx]

## 4. Sensitivity Analyses: Results

### a. Mendelian Randomization Sensitivity Analyses

All Mendelian randomization sensitivity analyses using summary data gave consistent results to the main Mendelian randomization analysis using individual participant data with the PRS for BMI, albeit with less precision and with a tendency to be closer to the null.

We estimated from the random-effects inverse variance weighted (IVW) analysis that a one kg/m^2^ increase in BMI caused a reduction in QALYs per year (-0.55% of a QALY, 95% CI: -0.69% to -0.41%, P value = 2.1x10^-15^) and an increase in total healthcare costs per year (£37.61, 95% CI: £29.09 to £46.14, P value = 5.1x10^-18^). There was little evidence of pleiotropy from Egger regression for any outcome, as Egger constant P values were 0.13 and 0.19 for QALYs and total healthcare costs, respectively. However, there was evidence of heterogeneity in SNP effects as P values for heterogeneity from Cochran’s Q value were <0.001 for both outcomes. See **S3 Table** for all Mendelian randomization sensitivity analysis results using summary data.

### b. Sex and Age Specific Analyses

There was little evidence of sex-specific effects in the main or age- or BMI-specific Mendelian analysis, **S4-7 Tables** have results split by sex.

From the Mendelian randomization analyses of categorical age, the estimated effect of a one kg/m^2^ increase in BMI on QALYs per year was -0.33% of a QALY per year (95% CI: -0.59% to -0.074%, P value = 0.01) for participants aged less than 50 years, and -1.04% of a QALY per year (95% CI: -1.47% to -0.60%, P value = 2.7x10^-6^) for participants aged 65 years or more. The estimated effect on total healthcare costs per year was £17.72 (95% CI: £4.05 to £31.39, P value = 0.01) for participants aged less than 50 years, and £78.46 (95% CI: £50.06 to £106.87, P value = 6.2x10^-8^) for participants aged 65 years or more. See **S4 Table** for full results by sex and age category.

There was additional statistical evidence for an interaction between BMI and age for all outcomes in the main Mendelian randomization analysis, as P values for age-BMI interaction terms were <0.001 for all outcomes, see **S5 Table**.

### c. Testing for non-linear effects of BMI

There was evidence for differences in the effect of a one kg/m^2^ increase in BMI on QALYs in different PRS-free BMI categories. The estimated effect of a one kg/m^2^ increase in BMI on QALYs per year was -0.22% of a QALY (95% CI: -0.54% to 0.10%, P value = 0.19) for the normal weight category (PRS-free BMI < 25 kg/m^2^), -0.68% of a QALY (95% CI: -0.90% to -0.46%, P value = 1.1x10^-9^) for the overweight category (PRS-free BMI ≥ 25 kg/m^2^ and < 30 kg/m^2^), and -0.81% of a QALY (95% CI: -1.02% to -0.60%, P value = 5.3x10^-14^) for the obese category (PRS-free BMI ≥ 30 kg/m^2^).

However, there was little evidence for differences in the effect of a one kg/m^2^ increase in BMI on total healthcare costs in different PRS-free BMI categories. We estimated that a one kg/m^2^ increase in BMI increased annual total healthcare costs by £29.38 (95% CI: £9.22 to £49.53, P value = 0.004) in the normal weight category, £45.28 (95% CI: £32.44 to £58.13, P value = 4.9x10^-12^) in the overweight category, and £43.94 (95% CI: £30.56 to £57.31, P value = 1.2x10^-10^) in the obese category.

Estimates for the effect of a one kg/m^2^ increase of BMI on all outcomes by age and PRS-free BMI categories together and by sex are available in **S4 Table**.

### d. Non-Linear Effects of BMI

There was evidence of non-linearity in the effect of BMI on QALYs from using VWLS across Mendelian randomization estimates from 50 quantiles of genetic-free BMI, **Fig A in S4 Text**. The estimated effect of one kg/m^2^ increase in BMI on QALYs per year changed by -0.055% of a QALY (95% CI: -0.082% to -0.027%, P < 0.0001) per one kg/m^2^ increase in BMI from the linear VWLS model, i.e. the effect grew more negative as BMI increased.

However, there was little evidence of non-linearity in the effect of BMI on total healthcare costs, **Fig B in S4 Text**; the estimated effect of a one kg/m^2^ increase in BMI on total healthcare costs increased by £1.43 (95% CI: -£0.41 to £3.26, P = 0.13) per one kg/m^2^ increase in BMI form the linear VWLS model. The cubic VWLS models showed similar non-linearity in the QALY estimates, but not the total health cost estimates, although this may be due to a lack of power to detect the effects. See **S6 Table** for results from each quantile of PRS-free BMI, and **S7 Table** for the linear and cubic VWLS results. **S2 Table** and **S3 Table** show the estimated effect of a one kg/m^2^increase in BMI on each outcome against the mean PRS-free BMI for each quantile of PRS-free BMI, with the cubic VWLS results plotted as a line. **Fig 3** and **Fig 4** in the main text show just the cubic VWLS results, with a confidence interval.


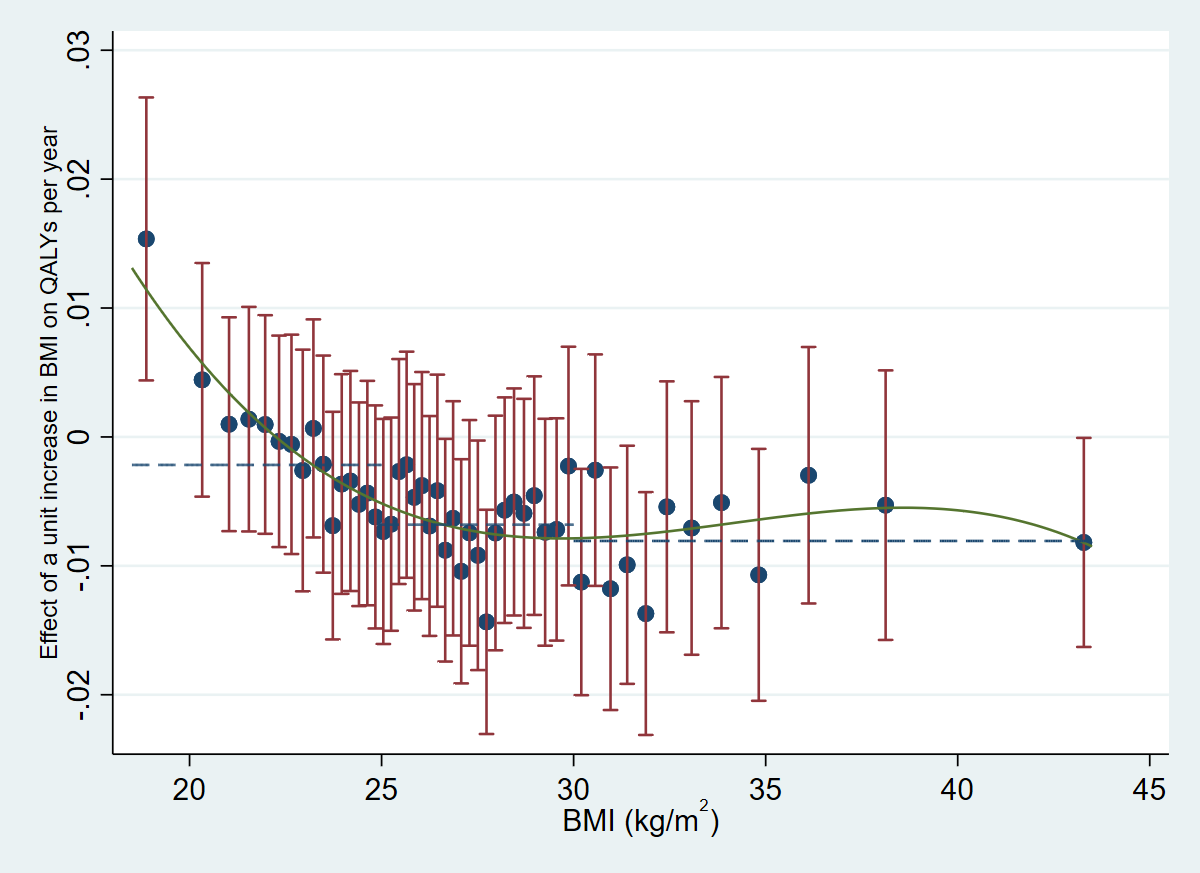


**Fig A:** The estimated effect of a one kg/m^2^ increase in BMI on average QALYs per year for each quantile of PRS-free BMI.

**Legend**: The solid green line indicates the trend line using cubic variance-weighted least squares. The dashed navy lines indicate the PRS-free BMI category specific estimates from the main Mendelian randomization analysis. The effect estimate for each quantile and its 95% confidence interval is represented by the blue points and red vertical lines. BMI = body mass index, PRS = polygenic risk score, QALY = quality-adjusted life year.


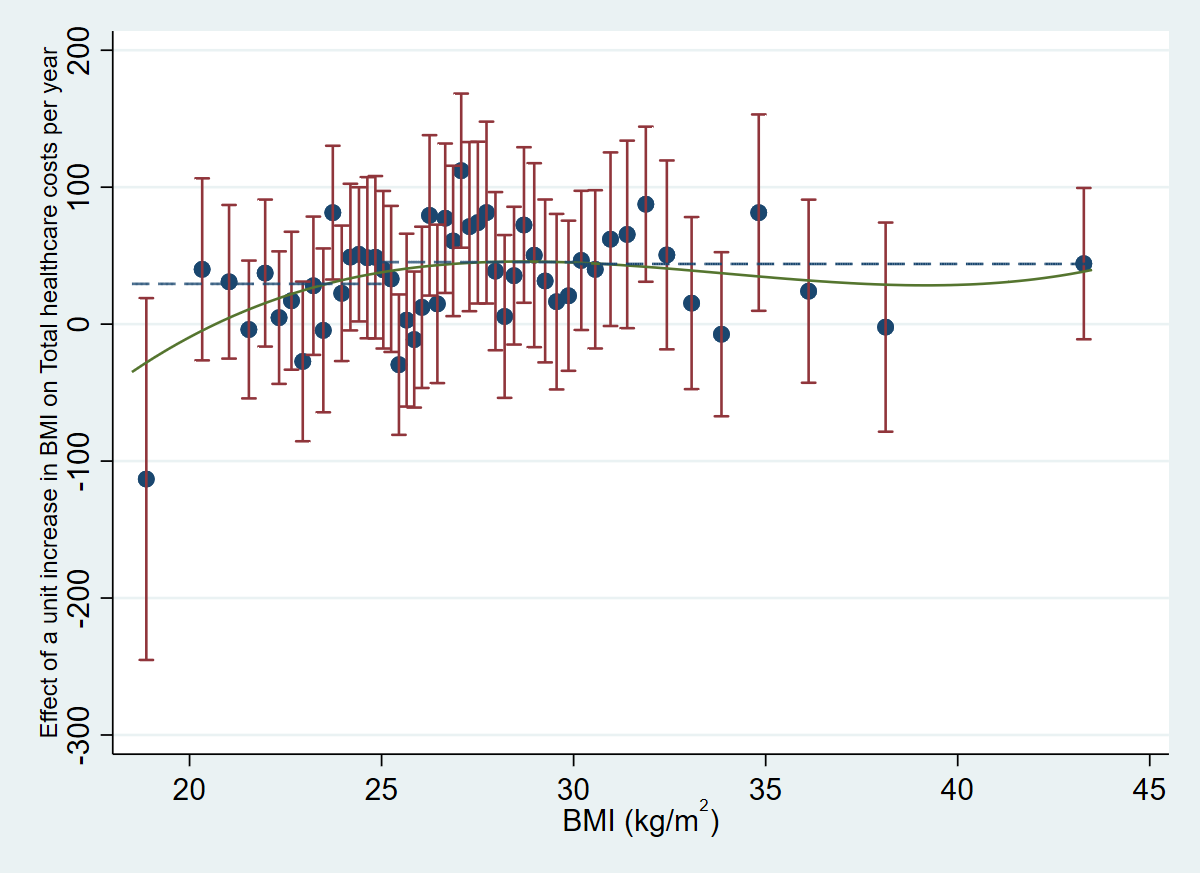


**Fig B**: The estimated effect of a one kg/m^2^ increase in BMI on average total healthcare cost per year for each quantile of PRS-free BMI.

**Legend**: The solid green line indicates the trend line using cubic variance-weighted least squares. The dashed navy lines indicate the PRS-free BMI category specific estimates from the main Mendelian randomization analysis. The effect estimate for each quantile and its 95% confidence interval is represented by the blue points and red vertical lines. BMI = body mass index, PRS = polygenic risk score, QALY = quality-adjusted life year.

### e. Within-Family Mendelian Randomization Analysis

Point estimates from the within-family Mendelian randomization analyses were consistent with the main Mendelian randomization analyses, but much less precise. We estimated in the within-family Mendelian randomization that a one kg/m^2^ increase in BMI caused a change of -0.48% of a QALY per year (95% CI: -1.30% to 0.35%, P value = 0.26), an increase of £6.11 in total healthcare costs per year (95% CI: -£49.27 to £61.49, P value = 0.83). The multivariable adjusted within-family analyses were consistent with the within-family Mendelian randomization analyses, but much more precise. See **S8 Table**.

### f. Accounting for uncertainty in the QALY predictions

Accounting for the uncertainty in the predicted QALYs did not change the results from the main Mendelian randomization or multivariable adjusted analyses, although the standard errors of the effect estimates increased. In the main Mendelian randomization analyses of QALYs, the standard error increased from 0.00084 when assuming an R^2^ of 100% in the Sullivan regression of utilities on health conditions (the main analysis), to 0.00115 when assuming an R^2^ of 0% in the Sullivan regression (taking death into account), an increase of 38%. Assuming R^2^ values of 75%, 50% and 25% gave standard errors of 0.00091, 0.00100 and 0.00107, increases of 9%, 20% and 27% respectively. Predicting QALYs without accounting for death gave very similar results to when accounting for death. See **S9 Table**.

Given the relatively small increase in standard errors when assuming a reasonable amount of prediction of health conditions for health-related quality of life, we did not consider it necessary to account for uncertainty in prediction of QALYs in the main analyses.

### g. Predicting health-related quality of life with limited health conditions

Predicting health-related quality of life using only the four health conditions generally used in simulation models as intermediates between BMI and cost-effectiveness captured far less effect of BMI on QALYs than using all 240 health conditions. Using this set of limited health conditions, from the main Mendelian randomization analysis we estimated that a one kg/m^2^ increase in BMI changed QALYs by -0.16% of a QALY (95% CI: -0.22% to -0.09%, P value = 4.3x10^-7^), as compared to a change of -0.65% of a QALY (95% CI: -0.81% to -0.49%, P value = 1.2x10^-15^) using all health conditions, see **S10 Table**.
