# Supplementary material for: Long-term cost-effectiveness of interventions for obesity: A mendelian randomisation study
Source: PLoS Med. 2021 Aug 27;18(8):e1003725. doi: 10.1371/journal.pmed.1003725 (PMC8437285; doi:10.1371/journal.pmed.1003725)
Supplement: Attachment — Submitted filename: Response to peer review.docx [file pmed.1003725.s025.docx]

-----------------------------------------------------------

Requests from the editors:

1. Data availability statement: Please provide the links (Github or other) for the code underlying the study. Please provide the access links for the UK Biobank data.

**The link to the code is already available, we have added a link to UK Biobank (2.9 Data and Code availability):**

***“The empirical dataset will be archived with UK Biobank and made available to individuals who obtain the necessary permissions from the study’s data access committees, with data accessible from https://www.ukbiobank.ac.uk/. The code used to clean and analyse the data is available here: https://github.com/sean-harrison-bristol/Robust-causal-inference-for-long-term-policy-decisions”***

2. Financial disclosure: Please slightly revise the final sentence of this section to read “The funders had no role in study design, data collection and analysis, decision to publish, or preparation of the manuscript”

**Added (Acknowledgements).**

3. Title: Please revise your title according to PLOS Medicine's style. Your title must be nondeclarative and not a question. It should begin with main concept if possible. "Effect of" should be used only if causality can be inferred, i.e., for an RCT. Please place the study design ("A randomized controlled trial," "A retrospective study," "A modelling study," etc.) in the subtitle (ie, after a colon).

**We have changed the title to:**

***“Long term cost effectiveness of interventions for obesity: a Mendelian randomization study”***

4. Abstract: Please structure your abstract using the PLOS Medicine headings (Background, Methods and Findings, Conclusions).

**Added (Abstract)**

5. Abstract Background: Provide the context of why the study is important. The final sentence should clearly state the study question.

**Added (Abstract):**

***“The prevalence of obesity has increased in the UK, and reliably measuring the impact on quality of life and the total healthcare cost from obesity is key to informing the cost-effectiveness of interventions that target obesity, and determining healthcare funding. Current methods for estimating cost effectiveness of interventions for obesity may be subject to confounding and reverse causation. The aim of this study is to apply a new approach using Mendelian randomisation for estimating the cost-effectiveness of interventions that target body mass index (BMI), which may be less affected by confounding and reverse causation than previous approaches.”***

6. Abstract: Please clarify this phrase: “When considering only health conditions usually considered in previous studies...”

**Changed (Abstract):**

***“When considering only health conditions usually considered in previous cost-effectiveness modelling studies (cancer, cardiovascular disease, cerebrovascular disease and type 2 diabetes)…”***

7. Abstract: Methods and Findings: Please provide more details of the study design and the main outcome measures before presenting the findings.

**Added (Abstract, underlined text is new, struckthrough text has been removed):**

***“We estimated health-related quality-adjusted life years (QALYs) and both primary and secondary healthcare costs for 310,913 men and women of white British ancestry aged between 39 and 72 years in UK Biobank between recruitment (2006 to 2010) and 31 March 2017. We then estimated the causal effect of differences in BMI on ~~quality-adjusted life years (~~QALYs~~)~~ and total healthcare costs using Mendelian randomization ~~in 310,913 men and women of white British ancestry aged between 39 and 72 years in UK Biobank, and applied our results to policy-relevant questions~~. Finally, we used simulations to estimate the likely effect on BMI of policy relevant interventions for BMI, then used the Mendelian randomization estimates to estimate the cost-effectiveness of these interventions.”***

8. Abstract: Methods and Findings: In the last sentence of the Abstract Methods and Findings section, please describe the main limitation(s) of the study's methodology.

**Added (Abstract):**

***“******The main limitations of this approach are that Mendelian randomization relies on assumptions that cannot be proven, including the absence of directional pleiotropy and that genotypes are independent of confounders.*** ***Large sample sizes are required for sufficient statistical power.”***

9. Abstract: Conclusions: Please address the study implications without overreaching what can be concluded from the data; the phrase "In this study, we observed ..." may be useful.

**Added (Abstract, underlined text is new):**

***“Mendelian randomization can be used to estimate the impact of interventions on quality of life and healthcare costs. We observed that the effect of increasing BMI on health-related quality of life is much larger when accounting for 240 chronic health conditions, compared with only a limited selection. This means that previous cost-effectiveness studies have likely underestimated the effect of BMI on quality of life, and therefore the potential cost-effectiveness of interventions to reduce BMI.”***

10. Author Summary: At this stage, **we ask that you include a short, non-technical Author Summary** of your research to make findings accessible to a wide audience that includes both scientists and non-scientists. The Author Summary should immediately follow the Abstract in your revised manuscript. This text is subject to editorial change and should be distinct from the scientific abstract. Please see our author guidelines for more information: <https://journals.plos.org/plosmedicine/s/revising-your-manuscript#loc-author-summary>

**Added (Author Summary):**

***“Why was this study done?***

***•	The prevalence of obesity has increased in the UK, and reliably measuring the impact on quality of life and the total healthcare cost from obesity is key to informing the cost-effectiveness of interventions that target obesity, and determining how much additional healthcare funding may be required should the trend of increasing obesity continue.***

***•	Current methods of examining cost effectiveness of interventions for obesity may be subject to confounding and reverse causation, and previous studies also typically only use a limited number of health conditions to estimate the effects of BMI on quality of life, potentially underestimating the effects of BMI.***

***•	The aim of this study is to elucidate a new approach using Mendelian randomisation for estimating the cost-effectiveness of interventions that target body mass index (BMI), which may be less affected by confounding and reverse causation than previous approaches***

***What did the researchers do and find?***

***•	We estimated the causal effect of differences in BMI on quality-adjusted life years (QALYs) and total healthcare costs using Mendelian randomization, used simulations to estimate the likely effect on BMI of policy relevant interventions for BMI, then estimated the cost-effectiveness of these interventions.***

***•	A unit increase in BMI was estimated to decrease QALYs by 0.65% of a QALY per year and increase annual total healthcare costs by £42.23 per person.***

***•	Compared to no intervention and over 20 years, we estimated that for people aged 40-69 years in England or Wales with a BMI over 35 kg/m2 receiving laparoscopic bariatric surgery would have, on average, an increase of 0.92 QALYs and a decrease in total healthcare costs of £5,096 per person.***

***•	We estimated that restricting volume promotions for high fat, salt and sugar products would, across the 21.7 million adults aged 40 to 69 years in England and Wales, increase QALYs by 20,551 per year and decrease total healthcare costs by £137 million per year.***

***•	We estimated that between 1993 and 2017 in England and Wales, the increase in BMI of people aged 40 to 69 years led to a decrease of 1.13% of a QALY per year and an increase in annual healthcare costs of £69 per person.***

***What do these findings mean?***

***•	Mendelian randomization can be used to estimate the impact of interventions on quality of life and healthcare costs and is likely less biased than existing observational methods.”***

11. Throughout: Please use square brackets for in-text citations. Please include line numbers with your revised document.

**Changed (throughout)**

12. Methods: Please ensure that the study is reported according to the STROBE guideline, and include the completed STROBE checklist (or the most appropriate checklist for your study) as Supporting Information. When completing the checklist, please use section and paragraph numbers, rather than page numbers. Please add the following statement, or similar, to the Methods: "This study is reported as per the Strengthening the Reporting of Observational Studies in Epidemiology (STROBE) guideline (S1 Checklist)."

**Changed (2.9 Data and Code Availability, Supplementary file with completed STROBE checklist):**

***“This study is reported as per the Strengthening the Reporting of Observational Studies in Epidemiology (STROBE) guideline (S1 Checklist).”***

13. Methods: Did your study have a prospective protocol or analysis plan? Please state this (either way) early in the Methods section.

a) If a prospective analysis plan (from your funding proposal, IRB or other ethics committee submission, study protocol, or other planning document written before analyzing the data) was used in designing the study, please include the relevant prospectively written document with your revised manuscript as a Supporting Information file to be published alongside your study, and cite it in the Methods section. A legend for this file should be included at the end of your manuscript.

b) If no such document exists, please make sure that the Methods section transparently describes when analyses were planned, and when/why any data-driven changes to analyses took place.

c) In either case, changes in the analysis-- including those made in response to peer review comments-- should be identified as such in the Methods section of the paper, with rationale.

**Changed (2.9 Data and Code Availability):**

***“This study did not have a prospective protocol or analysis plan: the analysis method was developed over the course of this study, and the policy analysis examples were considered before the method was finalised. No changes to the analysis were made from peer review comments.”***

14. Results: Please present results with 95% CIs and p values for all applicable analyses.

**95% CIs and P values are now given for all applicable analyses in the manuscript and supplement. Note that P values were not given for the policy analyses: their methodology (using simulations to give the median value for the main result and the 95% confidence interval representing the 2.5 and 97.5 simulation percentiles) does not produce a standard error, and so calculation of a Wald ratio is not possible. One-sided P values can be produced by counting the number of simulation results below/above 0, but in most analyses the P value would be 0. As such, we feel it would be better to not present P values for the policy analyses, given the they would reflect a different methodology to all other P values presented and provide little additional information above the 95% confidence intervals.**

15. Discussion: Please present and organize the Discussion as follows: a short, clear summary of the article's findings; what the study adds to existing research and where and why the results may differ from previous research; strengths and limitations of the study; implications and next steps for research, clinical practice, and/or public policy; one-paragraph conclusion.

**We have edited the discussion as below. We have included the “Implications and next steps for research, clinical practice, and/or public policy” second because we have an extensive section on limitations of the study, and this section fits better after the first paragraph. We are happy to change this at the editor’s discretion (Discussion, underlined text is new, struckthrough text has been removed):**

1. A short, clear summary of the article's findings

***“In this study, we have shown that cost effectiveness of clinical and policy interventions can be estimated using Mendelian randomization. We estimated the effect of a unit increase in BMI on average QALYs and total healthcare costs per year in UK Biobank, which showed that increasing BMI is detrimental to both QALYs and healthcare costs ~~in people with overweight and obesity~~. The effect ~~estimates were relatively stable once BMI had reached 25 kg/m2~~ of an increase BMI on healthcare costs and QALYs was relatively stable for BMI values above 25 kg/m^2^, implying that the expected ~~detrimental~~ effect of a~~n increase~~ change in BMI is very similar whether a person has a BMI considered overweight or obese. ~~whether a person has overweight or obesity, and, conversely, a reduction in BMI is similarly beneficial.~~ We used these estimates to show that bariatric surgery and the restriction of volume promotions for HFSS products are likely cost-effective relative to a “no intervention” comparator (net monetary benefit of £13,936 over 20 years) and estimated the costs of the increase to BMI over time (a decrease of 1.13% of a QALY and increase of £69 of annual healthcare costs per person) and having ~~overweight and obesity~~ a BMI above 25 kg/m^2^ in 2017 (a decrease of 3.73% of a QALY and increase of £230 of annual healthcare costs per person). ~~We emphasise that all cost-effectiveness estimates are relative to a “no intervention” comparator.~~”***

2. Implications and next steps for research, clinical practice, and/or public policy

***“We have demonstrated how Mendelian randomization can be useful for estimating the impact on quality of life and healthcare costs of either an exposure or intervention that is difficult, unethical or impossible to randomise (e.g. smoking, alcohol intake), or for interventions where long-term cost-effectiveness evidence from RCTs is rare or not generalisable (e.g. bariatric surgery). While in this study the conventional multivariable adjusted estimates not using genetic information were mostly similar to the Mendelian randomization estimates, this could be due to larger uncertainty in the Mendelian randomization estimates, and there is no guarantee that other exposures will be similar.*** ***We have also shown that considering more health conditions than cancer, cardiovascular disease, cerebrovascular disease and type 2 diabetes considerably increases the estimated effect of BMI on QALYs and healthcare costs, that laparoscopic bariatric surgery is likely to be cost-effective, and that the costs of population-level changes in BMI can be substantial.”***

3. What the study adds to existing research and where and why the results may differ from previous research

***“Previous studies examining the cost-effectiveness of interventions for obesity have used randomised controlled trials [9], cohorts [10–13], and decision analytic and related simulation models [10,12,14–18]. These studies estimated the impact on quality-adjusted life years (QALYs) and the total healthcare cost of different interventions, such as bariatric surgery, and thus estimated whether the intervention was likely to be cost-effective. Relative to existing methods, Mendelian randomization has longer follow-up, is cheaper and quicker, combines a more comprehensive set of outcomes, and is less likely to suffer from confounding and reverse causation. However, the disadvantages to Mendelian randomization for cost-effectiveness analysis are that it requires larger sample sizes, and we cannot be certain that the effects of life-long changes in BMI due to genetics will be comparable to changes induced by interventions. These relative strengths and limitations of the different approaches are summarised in Box 1.”***

4. Strengths and limitations of the study

***“Strengths and limitations***

***The estimates of the effect of BMI on QALYs and costs from Mendelian randomization are likely less biased by confounding and reverse causation than either cohort studies or decision analytic simulation models using observational effect estimates [20]. UK Biobank has many participants with comprehensive information about costs and disease states over many years. While the corresponding conventional multivariable adjusted estimates were generally consistent with the Mendelian randomization estimates for all outcomes, the Mendelian randomization estimates showed some detrimental effect of increasing BMI even in participants with BMI close to the top end of the normal weight category, while the conventional estimates did not, which could reflect bias in the conventional estimates.***

***This method of estimating the effect of a risk factor on QALYs and costs can be extended to other risk factors with causal genetic components, and also provide evidence for the causal effects of health conditions on healthcare costs and QALYs. This may be useful for health conditions that are strongly influenced by risk factors that affect other health conditions where the effect of the condition would otherwise be confounded by the risk factor, such as cardiovascular disease.***

***However, Mendelian randomization relies on assumptions that cannot be proven [20], as is the case with all types of instrumental variable analysis and other forms of observational policy evaluation. There was evidence for heterogeneity between SNPs for all outcomes, though in general the summary Mendelian randomization sensitivity estimates were consistent with the main estimates, and there was little evidence of directional pleiotropy from the MR Egger regression. As the outcomes were not biological, the exclusion restriction assumption (i.e. that any genetic variant affects the outcome only through the exposure) may not hold for all the genetic variants (i.e. that the genetic variant affects the outcome only through the exposure).***

***These estimates represent a lifetime exposure to a genetic influence on BMI, and thus cannot be interpreted directly as the expected effect of an intervention at a specific age. In general, as the age at which a person received an intervention increases, the effect estimates would likely reduce. This is because the mechanisms by which BMI affects health may be cumulative over time, and so even if BMI were lowered in older age, some residual detrimental effect of previously high BMI may remain. It is therefore likely that our estimates of the impact of BMI on costs and QALYs are best applied to population level interventions that aim to reduce BMI across all age groups. This limitation is also present in decision analytic simulation models of cost-effectiveness, though not RCTs or cohort studies. Our estimates may also underestimate the true effect as people in England and Wales now may have had larger BMI values earlier in life than previously, increasing the length of exposure to obesity. It is also the case that the Mendelian randomization estimates may be fully representative of interventions that target BMI, as these interventions will typically target more than just a change in BMI, including exercising more or improving diets. Therefore, the generalisability of our results to interventions for BMI will depend on how comparable the intervention is to causing a genetically determined difference in BMI.***

***For all policy examples, we require the stable unit treatment value assumption for causal inference, this assumption requires that genetic change in BMI is equivalent to a change in BMI by other means, e.g. by bariatric surgery or reducing Caloric intake of high fat, sugar and salt foods. This assumption is not testable. Mendelian randomization analyses can also be interpreted as estimates of a “local average treatment effect”, by assuming that changes in the genetic variants affecting BMI affect all participants in UK Biobank in the same direction (monotonicity). This is assumption also cannot be tested, and deviations from monotonicity could bias effect estimates.***

***The analyses accounting for QALY prediction error were consistent with the main analysis, although less precise. We predicted QALYs using data from Sullivan et al [36], as QALYs have not been previously estimated in UK Biobank. While this data is applicable to a UK population, this method only captures health-related quality of life, and therefore our QALY estimates do not include any non-health-related determinants of quality of life. This was unavoidable given the data available in UK Biobank, where only linked healthcare data was available beyond baseline (excepting the relatively small amount of data from follow-up visits): future studies repeatedly measuring quality of life directly may therefore provide more robust effect estimates. We also had to impute primary care costs and QALYs as only a limited section of UK Biobank had primary care data, which limited statistical power but was unlikely to have biased the results, rather, the complete case analysis would likely have been biased results, since the distribution of GP software systems allowing linkage of primary care data is unlikely to be random.***

***The healthcare costs were estimated from observed hospital episodes, drug prescriptions and appointments from primary care. Follow-up was two years shorter for secondary care costs than primary care costs, but as we averaged the costs this should not have materially affected the results. Additionally, we did not capture all healthcare costs as we did not have access to private healthcare costs not incurred in NHS settings, or data for emergency care or outpatient appointments (which are not linked to the UK Biobank cohort), and did not consider the cost of diagnostic tests in primary care, likely therefore under-estimating the total cost of increasing BMI. In contrast, participants in UK Biobank may have different access to healthcare than the country on average, which may have biased our estimates of the effect of BMI on costs. Finally, BMI may have interacted with the use of both state and private healthcare, potentially biasing the results in either direction.***

***In the policy analyses, we made several assumptions: that bariatric surgery had no effects on QALYs through anything other than its effect on BMI, including no perioperative mortality or side-effects (though complications of bariatric surgery on total healthcare costs up to 5 years was included in the cost of surgery); that the estimated BMI reduction from bariatric surgery would be maintained over 20 years; and that both UK Biobank and the Health Survey for England were representative of the population of England and Wales. These assumptions appear justifiable, as the average effect of bariatric surgery on QALYs over 20 years is likely relatively low, bariatric surgery has shown a consistent reduction in BMI up to 20 years [58,59], and the Health Survey for England is nationally representative [1,2].***

***However, despite its size, UK Biobank is not representative of the UK population as participants tend to be wealthier and healthier compared to the country on average [64]. It therefore likely that we have underestimated the true costs of BMI, as wealthier and healthier people may be more resistant to any detrimental effects of increased BMI. As obesity is more common in lower socioeconomic groups [65], our results indicate that obesity is likely a cause of inequalities in quality of life.***

***Although Mendelian randomization is likely to be less affected by confounding and reverse causality than conventional multivariable adjusted analyses, an important potential source of bias in these analyses is family-level effects. Recent evidence suggests that assortative mating and dynastic effects can lead to bias in Mendelian randomization effect estimates [56], though within-family Mendelian randomization studies can account for some of these biases. Our within-family sensitivity analyses showed that the effect of BMI on QALYs was consistent with the main analysis, though the effect of BMI on total healthcare costs was reduced. However, statistical power was limited in these analyses, and confidence intervals were wide. Additionally, there is evidence of a geographic structure in the UK Biobank genotype data that cannot be accounted for using adjustment for principal components, which may also have biased our analyses [66].”***

5. One-paragraph conclusion

***“~~5. Conclusion~~***

***Mendelian randomization can be used to estimate the effect of an exposure on quality of life and healthcare costs. We used this approach to estimate the cost effectiveness of interventions aimed at reducing BMI. This approach could be especially useful where it is difficult, unethical or impossible to randomise participants to an exposure such as obesity or for prevalent behaviours with adverse health impacts such as smoking or alcohol use, or where RCT evidence is rare for an intervention. Results from such studies are likely of benefit to both policy and the NHS. In future studies, we will use this method to assess the costs of different risk factors for poor health.***

***The effect of increasing BMI on health-related quality of life may be larger than previously thought, as decision analytic simulation models may underestimate the effect of BMI on QALYs by using only limited health conditions are intermediates.”***

16. References: Please use the "Vancouver" style for reference formatting, and see our website for other reference guidelines <https://journals.plos.org/plosmedicine/s/submission-guidelines#loc-references>

**Vancouver with square brackets is used throughout.**

17. Figures and Tables: **Please provide titles and legends for all figures and tables (including those in Supporting Information files).**

**Titles and legends added for all figures and tables in the main manuscript, and figures in the supplement, below the corresponding figure or table. Titles and legends for supplementary tables provided at the end of the supplement. All titles and legends are also provided as a supplementary file.**

18. Figures 2 and 3: Please note in the legend these points are shown with 95% CIs.

**Changed (figure titles and legends)**

19. Figure 4 and 5: Please note the dotted vertical lines in the legend. Please note the shaded area indicates the confidence interval (if accurate).

**Changed (figure titles and legends)**

Comments from the reviewers:

Reviewer #1: The authors report a Mendelian randomization study to assess the causal effect of higher BMI on QALYs and total health care costs, and to assess the cost-effectiveness of two interventions that lower BMI, including laparoscopic surgery and restriction of volume promotions for high fat, salt and sugar products, on QALYs and total health care costs. Genetic variants identified in genome-wide association studies for BMI are applied as instrumental variables in analyses of 310,913 participants of the UK Biobank. The authors find that each unit increase in BMI is causally associated with 0.0065 decrease in QALYs per person per year, and 42.23£ increase in total health care costs per person per year. They estimate that laparoscopic surgery over 20 years would lead to a total increase in QALYs of 0.92 per person and a decrease in total healthcare costs of 5,096£ per person. They also estimate the restricting volume promotions for high fat, sat and sugar products across 21.7 million adults in 40-69 years in England and Wales would increase QALYs by 20,551 per year and decrease health care costs by 137£ million per year.

Overall, the paper is very relevant for the readership and carefully written, and demonstrates the relevance of the Mendelian randomization method as a potential new adjunct for decision-making relating to health care policies. While there are multiple limitations and assumptions to the cost-effectiveness estimations and to the Mendelian randomization method itself, I consider that they are comprehensively and appropriately discussed in the paper. Thus, I'm pleased to recommend the paper to be accepted for publication.

**We thank reviewer #1 for their kind comments.**

Reviewer #2: The present manuscript aims at examining causal relationship between changes in BMI levels and quality of life and healthcare cost through Mendelian randomization (MR) approach using instrument variable regression analyses. The authors have demonstrated how available genetic datasets can be utilized for deriving causal inferences for cost effectiveness of public health interventions. This approach is immensely useful as it requires comparatively less resources and time a compared to other methods like trials and cohorts that are expensive to conduct and are more time consuming. However, the MR findings should be carefully interpreted as they depend on the robustness of the instrument variable (genetic proxy) of the exposure on causal mechanism being examined. The authors have described how they ruled out the bias of pleiotropy as well which is very crucial in MR analyses. The authors have used very robust instrument fr BMI using established GWAS loci while excluding recent studies that included UK Biobank population. Apart from examining the causal effect of causal effect of BMI on QALYs and healthcare costs, they examined the effect on MR results on different policies as well that are immensely informative for policy advocacy. The detailed methodology and the sensitivity analyses demonstrates authors thorough understanding of MR technicalities and their ability to utilize MR approach to answer research questions of public health relevance.

I have no specific queries or recommendations to revise the manuscript. This manuscript can be accepted in its present form.

**We thank reviewer #2 for their kind comments.**

Reviewer #3: This an exhaustive study, with an extended number of secondary and sensitivity analyses. The Mendelian Randomization analyses with individual level data and with summarized data are correctly applied.

The present work is relevant since it has the potential to inform policies that improve the quality of life and the healthcare costs by taking measures that reduce the BMI of the population.

Generally, I feel that the manuscript should be written in a clearer manner. In addition, I have other comments:

1. The results section is very dense and hard to follow in some parts. Authors might consider to re-write it a bit.

**We revised the manuscript for clarity and conciseness throughout.**

2. I do not understand why the QALYs units are sometimes expressed in % and sometimes in costs per year (pounds). Please clarify.

**The manuscript does not express QALYs as costs per year. In the manuscript, we estimate the change in QALYs and the change in healthcare costs from a unit increase in BMI. In the supplement, we also estimate QALY-costs, which are the total healthcare costs minus £20,000 multiplied by change in QALYs. This quantity allowed us to estimate the effect of a unit increase in BMI on the combined QALYs and healthcare costs: in the supplement and the supplementary tables, this quantity is labelled as “QALY-costs”.**

**We have added notes to all tables with QALY-costs in them, explaining what QALY-costs are.**

3. In “Policy Analyses, section d)”, authors say that if all participants with BMI >25kg/m2 had a BMI of 25kg/m2, the QALYs would be decreased. Is it correct like this? One would expect that if all people changed from overweight to normal weight, the quality of life would increase.

**We have clarified the sentence in question (QALYs are indeed reduced with higher BMI values):**

**“We estimated that, compared to if all people with a BMI above 25 kg/m^2^ aged 40 to 69 years in England and Wales in 2017 had a BMI of 25 kg/m^2^, the current BMI profile of England and Wales decreases QALYs by 3.73% per person…”**

4. Table 2 and Figures 2 and 3 have slightly different numbers for the overall results. Please double check.

**The results in figures 2 and 3, as well as all relevant supplementary tables, have been updated to include the imputation from which the results were derived, and the numbers have been fixed in the figures.**

5. I feel that a discussion of the results from a clinical point of view or comparing the results with previous studies is missing in the results section.

**We have included more in the discussion about previous studies, though with the caveat that the methods presented in this paper have not been, to our knowledge, used previously in this context (Discussion, 3^rd^ paragraph):**

***“Previous studies examining the cost-effectiveness of interventions for obesity have used randomised controlled trials [9], cohorts [10–13], and decision analytic and related simulation models [10,12,14–18]. These studies estimated the impact on quality-adjusted life years (QALYs) and the total healthcare cost of different interventions, such as bariatric surgery, and thus estimated whether the intervention was likely to be cost-effective. Relative to existing methods, Mendelian randomization has longer follow-up, is cheaper and quicker, combines a more comprehensive set of outcomes, and is less likely to suffer from confounding and reverse causation. However, the disadvantages to Mendelian randomization for cost-effectiveness analysis are that it requires larger sample sizes, and we cannot be certain that the effects of life-long changes in BMI due to genetics will be comparable to changes induced by interventions. These relative strengths and limitations of the different approaches are summarised in Box 1.”***

**We thank reviewer #3 for their comments.**

Reviewer #4: This paper describes a study that used Mendelian randomization to estimate the effect of high body mass on (monetized) quality of life and health care costs and estimate the economic merit of a few policy-relevant scenarios. The paper is eloquently written and uses innovative methods to address an issue of large public health relevance. I have a few questions for clarification but found this to be a strong study overall.

The study presents remarkable findings regarding the effect of BMI on outcomes that is not via the usually modelled major disease groups (which is of clear relevance to modellers like me). However, that effect seems related to the imputation of quality of life estimates for each participant, which was done using Sullivan's method, rather than from the Mendelian randomization. Is that correct?

The QALY percentages used to express the impact of changes in BMI on quality of life, are those percentage-points (with 100%, or 1 QALY, as basis), or relative percentages (with 'current'/prior QALY values as basis)?

Were life years lost accounted for in the QALYs? How - by dividing QALYs by 20 even if life was cut short? Was each death valued as 1 QALY lost per year till the end of follow-up (which would underestimate losses) or was it multiplied by life expectancy at the age of death (with what value?)? I would expect the former to be the case, consistent with the 20-year time horizon. Which of course means that while most or all of the costs of the interventions have been taken into account in these analyses, the benefits have been understated, and the true cost-effectiveness of interventions will have been underestimated.

In contrast, the assumptions regarding bariatric surgery, notably the assumption of no adverse side-effects and no peri-operative mortality, will have resulted in some degree of overestimation of the economic credentials of this intervention.

Does the Mendelian randomization approach produce realistic results, since most (if not all) interventions to reduce weight act via changes in diet or physical activity? Large as the results are, if they reflect purely the effect of extra body mass (and not also those of, say, lower sugar consumption or more physical activity) are they not likely underestimates of the true gains that can be expected from interventions?

Minor comments

Typo 'BNI' on page 12 of the supplementary file.

***“…****that effect seems related to the imputation of quality of life estimates for each participant, which was done using Sullivan's method, rather than from the Mendelian randomization. Is that correct?****”***

**Yes - the overall effects are dependent on the QALYs estimated using Sullivan’s method. We have added this limitation to the discussion (4. Discussion, Strengths and limitations):**

***“We predicted QALYs using data from Sullivan et al (36), as QALYs have not been previously estimated in UK Biobank. While this data is applicable to a UK population, this method only captures health-related quality of life, and therefore our QALY estimates do not include any non-health-related determinants of quality of life. This was unavoidable given the data available in UK Biobank, where only linked healthcare data was available beyond baseline (excepting the relatively small amount of data from follow-up visits): future studies repeatedly measuring quality of life directly may therefore provide more robust effect estimates.”***

**Note that while we have no reason to think that the Sullivan method wouldn’t be appropriate, the method of using Mendelian randomisation to estimate cost effectiveness presented in this paper just requires an estimate of QALYs, so other methods could be used in the future. Ideally, QALYs would be periodically recorded in the population of interest, obviating the need to use any particular method to estimate them. Note also that we include a sensitivity analysis where we include uncertainty when predicting QALYs for all participants (sensitivity analysis f).**

*“The QALY percentages used to express the impact of changes in BMI on quality of life, are those percentage-points (with 100%, or 1 QALY, as basis), or relative percentages (with 'current'/prior QALY values as basis)?”*

**The QALY percentages were percentage points. We’ve made this more explicit in the manuscript (Section 2.4, Quality-Adjusted Life Years):**

***“Throughout this manuscript, we report the change in the number of QALYs, either in whole numbers or percentage points, e.g. 0.65% of a QALY means 0.0065 QALYs.”***

*“Were life years lost accounted for in the QALYs?”*

**Deaths were included in the estimation of QALYs in UK Biobank, and so were included in the estimated effects of BMI on QALYs. The simulations were only designed to estimate the change in BMI in specific age groups and BMI categories: after this, the estimated age- and BMI- specific changes in BMI were multiplied by the Mendelian randomization derived effect estimates for QALYs (including deaths) and total healthcare costs.**

*“In contrast, the assumptions regarding bariatric surgery, notably the assumption of no adverse side-effects and no peri-operative mortality, will have resulted in some degree of overestimation of the economic credentials of this intervention.”*

**We added the assumptions of no peri-operative mortality from bariatric surgery and no adverse side-effects of bariatric surgery on QALYs to the discussion, although complications from bariatric surgery on total healthcare costs up to 5 years (based on available evidence from RCTs) were included in the cost of treatment (4. Discussion, Strength and limitations):**

***“In the policy analyses, we made several assumptions: that bariatric surgery had no effects on QALYs through anything other than its effect on BMI, including no perioperative mortality or side-effects (though complications of bariatric surgery on total healthcare costs up to 5 years was included in the cost of surgery); that the estimated BMI reduction from bariatric surgery would be maintained over 20 years; and that both UK Biobank and the Health Survey for England were representative of the population of England and Wales. These assumptions appear justifiable, as the average effect of bariatric surgery on QALYs over 20 years is likely relatively low, bariatric surgery has shown a consistent reduction in BMI up to 20 years (31,32), and the Health Survey for England is nationally representative (1,2).”***

*“Does the Mendelian randomization approach produce realistic results, since most (if not all) interventions to reduce weight act via changes in diet or physical activity?”*

**This is an interesting question that is difficult to answer. Different genetic profiles will affect BMI through different mechanisms, including through different activity and hunger levels, as well as diets and body composition, so it is probably fairest to suggest that the Mendelian randomisation results reflect an average change due to a change in BMI from a variety of sources across a population.**

**It is likely that interventions that target exercise will have different long-term effects to those that target diets, but RCTs of sufficient duration (or retention) are unlikely to be conducted to give definitive answers. Whether the Mendelian randomization estimates would be smaller or larger than any long-term RCTs is unknown, and would, we imagine, depend on the nature of the intervention in question and how much it affects systems other than BMI. It is certainly plausible that interventions aiming to cause a sustained increase in activity levels would have benefits beyond decreasing BMI. In theory, multivariable Mendelian randomization including both BMI and physical exercise could be performed to give more targeted answers for specific interventions, if sufficient data and genetic variants are available.**

**We have added some thoughts to the discussion (Discussion, Strengths and limitations):**

***“It is also the case that the Mendelian randomization estimates may be fully representative of interventions that target BMI, as these interventions will typically target more than just a change in BMI, including exercising more or improving diets. Therefore, the generalisability of our results to interventions for BMI will depend on how comparable the intervention is to causing a genetically determined difference in BMI.”***

*“Typo 'BNI' on page 12 of the supplementary file.”*

**The typo on page 12 of the supplement has been corrected, thank you.**

**We thank reviewer #4 for their comments.**
