# Supplementary material for: Long-term cost-effectiveness of interventions for obesity: A mendelian randomisation study
Source: PLoS Med. 2021 Aug 27;18(8):e1003725. doi: 10.1371/journal.pmed.1003725 (PMC8437285; doi:10.1371/journal.pmed.1003725)
Supplement: Attachment — Submitted filename: Response to peer review.docx [file pmed.1003725.s026.docx]

------------------------------------------------------------

Requests from Editors:

1. Thank you for clarifying the methodology presentation in the abstract. We feel that this is important for understanding the study. However, while the abstract is thoroughly presented, we request that you please revise to shorten the abstract to no more than 500 words.

-We suggest summarizing the presentation of the results for the simulations of BMI-targeting interventions, presenting fewer details, such as:

“We estimated that both laparoscopic bariatric surgery among individuals with BMI greater than 35 kg/m2, and restricting volume promotions for high fat, salt, and sugar products would increase QALYs and decrease total healthcare costs.”

-We suggest similarly summarizing or removing the results describing the decrease in QALY and increase in costs estimated for the increases in BMI between 1993 and 2017, and the decreases in QALYs and costs associated with universal BMI below 25 kg/m2:

“Between 1993 and 2017 in England and Wales, the increase in BMI of people aged 40 to 69 years led to a decrease of 1.13% of a QALY per person per year (95% CI: 0.90% to 1.38%) and an increase in annual healthcare costs of £69 per person (95% CI: £53 to £84).

Compared to if all people with a BMI above 25 kg/m2 aged 40 to 69 years in England and Wales in 2017 had a BMI of 25 kg/m2, QALYs are decreased by 580,494 in total per year (95% CI: 457,907 to 717,691) and annual healthcare costs are increased by £3.58 billion (95% CI: £2.75 billion to £4.34 billion).“

-We suggest removing the following sentence from the limitations: “Sample sizes typically must be larger to achieve the same level of statistical power as in corresponding observational studies.” as this seems to be a very general limitation.

**Abstract now <500 words.**

2. Methods: Line 338: Please double check the link referring the reader to information on patient/public involvement, the second link does not seem to work.

**Link deleted, the first link will suffice.**

3. References: Please update references 23 and 54 with the complete information.

**References updated.**

**--Please consider either making a “response to peer review” document mandatory when resubmitting (i.e. make it so the system doesn’t accept a resubmission without such a document), or accepting a response in the cover letter, when the peer review in question only contains comments from the editors.**
